# Enzyme promiscuity in the field of synthetic biology applied to white biotechnology: Opportunities and weaknesses

**DOI:** 10.1016/j.bidere.2025.100026

**Published:** 2025-05-14

**Authors:** Thibault Malfoy, Ceren Alkim, Jean Marie François

**Affiliations:** a Toulouse Biotechnology Institute (TBI), Université de Toulouse, CNRS, INRA, INSA, 135 Avenue de Rangueil, F-31077, Toulouse, France; b Toulouse White Biotechnology Center (TWB, UMS INRAE-INSA-CNRS), 135 avenue de Rangueil, F31077, Toulouse, France

**Keywords:** Enzyme promiscuity, Repair enzyme, Underground metabolism, Synthetic biology, White biotechnology, Bioeconomy

## Abstract

White biotechnology stands as a major sustainable alternative to address pressing environmental issues arising from our heavy dependence on petrochemical synthesis. However, reaching this goal, both technologically and economically, will take time, resources and money. A major reason is within the biological system itself, as it has evolved into a bow-tie structure in which carbon and energy are converted, via highly regulated, complex and interconnected metabolic networks, into cellular components for growth and homeostasis. This objective is fundamentally at odds with that of biotechnology, which aims to convert carbon and energy into bioproducts. Engineering of microorganism using systems and synthetic biological systems tools has been developed to provide a compromise between these two objectives. However, these genetic and metabolic interventions have revealed often unexpected physiological behaviors, in part due to the fact that a large proportion of metabolic enzymes are catalyzing other reactions than those for which they were evolved. While this promiscuity is the source of an underground metabolism that can prove very advantageous in building high-performance production routes, it is also responsible for loss of yield and production due to metabolic disturbances, negative cross-talks between natural and heterologous pathways as well as it is at the onset of metabolic damages. Identifying these promiscuous enzymes and thus anticipating their opportunities or weaknesses in engineering microbial cell factories for bioproduction is a major challenge in order to improve their performance. It is foreseen that machine learning tools operating on databases continuously fed by genetic, metabolic, enzymatic and fermentation processes data can help to overcome these challenges and provide a better understanding of the physiological functioning of the microbial system.

## 1 Challenges in white biotechnology and how synthetic biology could contribute to it

**Fig. 1:**
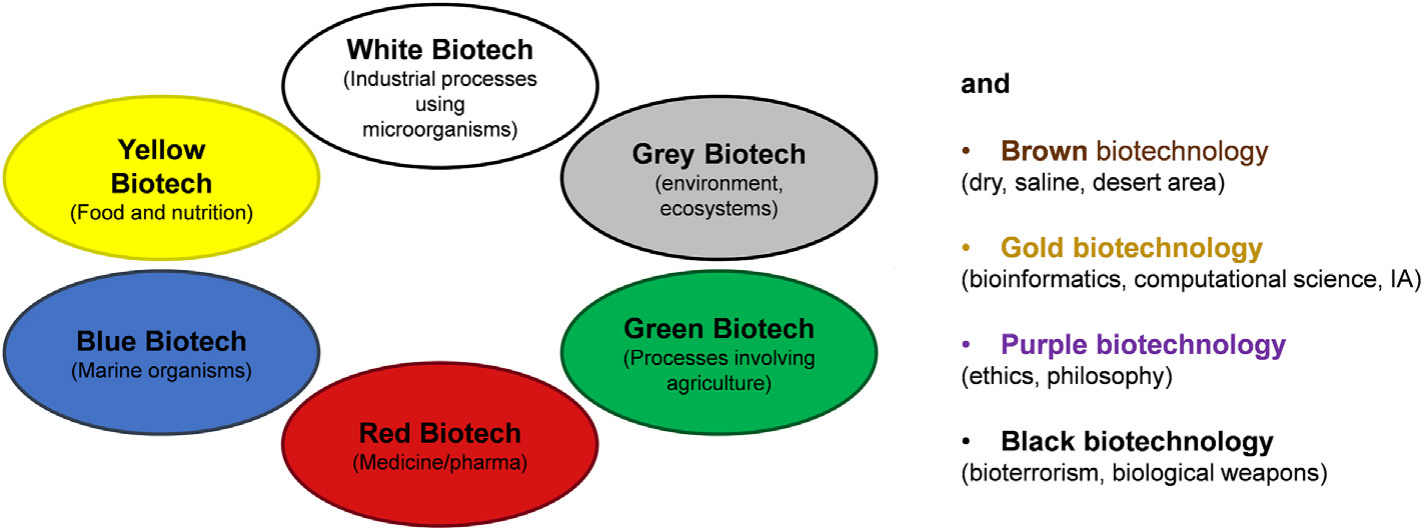
Branches of biotechnology as identified by color code.

**Fig. 2:**
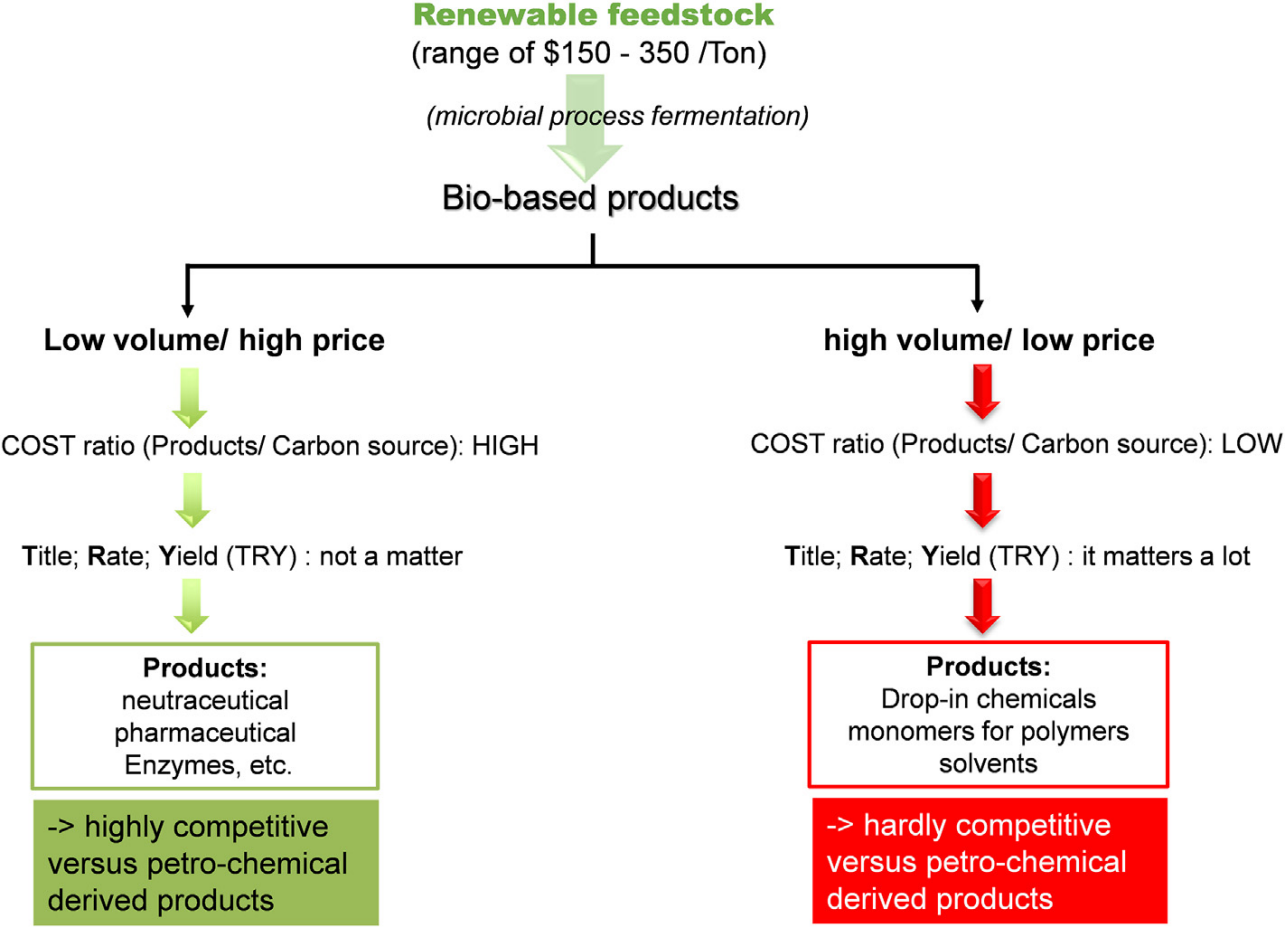
Typology of bio-based products and their relevance towards similar petro-chemical derived products.

The term “biotechnology” has been originally coined in 1919 by a Hungarian agricultural engineer Karoly Ereky [1]. According to OCDE definition (https://stats.oecd.org/glossary/detail.asp?ID&equals;219), biotechnology is defined as the application of science and technology to living organisms and their parts, products or models, to modify living or non-living materials for the production of knowledge, goods and services. It comprises 10 branches or divisions that are recognized by a code color (Fig. 1). Among them, white biotechnology also termed industrial biotechnology is the oldest branch, having its roots in traditional fermentation of beer, wine and fermented food. It is nowadays better defined as the biotransformation of raw material –renewable and nonedible-carbon feedstock into a variety of bio-based products and goods by the activity of natural or genetically modified microorganisms, or enzymes derived from them, in a confined environment, *i.e.* in closed bioreactors. Broadly speaking, two typologies of bio-based products can be manufactured through biotechnological processes (Fig. 2): (i) commodity products, also termed drop-in chemicals, that are produced in very large volume and have a low market value (around $ 1.0/kg), such as ethanol, 1,3-propanediol, 1,4- butanediol, 3-hydroxypropionic acid, etc. [2] and (ii) specialty products that are produced in low volume with a high market value, at least $ 10/kg up to several thousand dollars per kg, such as antibodies, vaccinees, therapeutic products, etc. Therefore, the consideration of the three key metrics-**t**itre (g/L), **r**ate (g.h^−1^ L^−1^) and **y**ield (g of product per g of feedstock/biomass) - symbolized by the nickname ‘TRY’ which contributes to the techno-economic evaluation of the bioprocess will be considered differently between the two typologies [3,4]. Clearly, TRY needs to be as high as possible for the bioproduction of chemicals and related products such as biopolymers, bioplastics, etc., in order to compete with their petrochemical counterparts, as the margin between the cost of the raw material (around $0.15 - $0.4/kg) and the biobased products is too small for its biobased products to be competitive at the moment. In addition, while the petrochemical factories are well mature and already well depreciated, biotechnology factories are still very young quite immature but there's plenty room for improvement [5].

In spite of these economic constraints, industrial biotechnology is arguably one of the most important branches of biotechnology that could address pressing environmental issues arising from our slave dependency to fossil fuels [6,7]. However, this ambitious goal cannot be achieved by exploiting microorganisms solely on the basis of their intrinsic metabolic characteristics, because they are not sufficiently profitable in industrial terms, and more importantly, the most desired drop-in chemicals are not products of their natural metabolic activity. Metabolic engineering combined with synthetic biology and evolutionary engineering provide tools and strategies to address these limitations, enabling the development of microbial cell factories with the desired technical trait able to produce a large portfolio of non-natural drop-in chemicals [[8], [9], [10], [11]]. However, despite the remarkable advances in systems and synthetic biology applied to white biotechnology over the last two decades, which has been documented in many excellent reviews [5,[12], [13], [14], [15], [16], [17], [18]], the success stories of high volume/low price bulk chemicals produced by microbial processes fermentation from non-edible biomass that have reached a commercial scale can be counted on the fingers of one hand [19]. Apart from the still low cost of fossil oil, and that the cost of carbon feedstock is unfortunately linked to oil prices, which represent some economic constraints unfavorable to the emergence of industrial biotechnology, developing new cell factories that meet the economic requirements of industrial-scale production is a real challenge, requiring more than 10 years and millions of dollars in research and development. The reasons lie in the living system itself, which is highly complex, unpredictable and unstable, in the sense that it can evolve and lose the new technical properties it has been ingeniously introduced to over time and through use [20,21].

In this opinion paper, the authors wish to discuss a particular aspect that contributes to the complexity and unpredictability of harnessing biological systems through their metabolic network for bioproduction purposes. It deals with the promiscuity of enzymes, their potential importance in the emergence of underground metabolism (see explicit definition in section 4) leading to interactions with normal pathways. These interactions raising from enzyme promiscuity has been often ignored in the design of bioproduction pathways and are only identified during the “learn” phase of the Design Build Test Learn (DBTL) cycle for microbial engineering systems [22,23]. Anticipating such interactions at the beginning of the design cycle could be highly beneficial because on the one hand, enzyme promiscuity can offer opportunities in building and optimizing cell factories and on the other hand, these reactions may lead to unexpected disruptive effects on the metabolic networks, causing loss of bioproduction efficiency. This double-sided face of promiscuity will be highlighted in this review through several cases studies.

## 2 Enzymatic promiscuity: how is it defined, can it be predicted and quantified?

A clear definition of enzyme promiscuity is still a matter of discussion [[24], [25], [26], [27], [28], [29]]. It entails two interrelated criteria, which is catalytic promiscuity, *i.e.* an enzyme that catalyzes more than one reaction, and substrate ambiguity, *i.e*. an enzyme with broad substrate specificity, both criteria can co-occur for a single enzyme such as D-malate dehydrogenase, which has a dehydrogenase activity on different substrates like *m*-tartrate, isopropylmalate, oxaloacetate and 2-oxo-4-hydroxybutyrate (T.Malfoy & J. François, unpublished data) and also displays an oxidative decarboxylating activity of D-malate [30]. To this general definition, Khersonsky & Tawfik [31] further proposed to restrict the term “promiscuity” to those enzymes that are characterized by a catalytic efficiency (k_cat_/K_M_) on a new (non-natural) substrate that is 100 to over 10,000 times lower than on their “physiological” substrate for which they have evolved. This complementary definition would likely exclude enzymes with ‘broad specificity’ albeit exhibiting similar catalytic efficiency like several amino-acids transaminases [32] unless they have been evolved to carry on a reaction on non-natural substrates such as the alanine transaminase encoded by *alaC* in *E. coli*, which has been evolved to a L-homoserine transaminase able to produce the non-natural metabolite 2-oxo-4-hydroxybutyrate (OHB) from L-homoserine [33]. This would also exclude moonlighting or multifunctional enzymes, which are enzymes that deserves two distinct functions, such as galactokinase in yeast that catalyzes galactose phosphorylation and meanwhile is part of the transcriptional regulation of *GAL* genes [34]. On the other hand, this restriction fits perfectly into the context of synthetic biology applied to industrial biotechnology since bioproducts of industrial relevance are obtained via synthetic metabolic pathways relying on engineering and evolution of enzymes with promiscuous activity [35,36].

Since evolution, and in particular that of metabolic pathways, has capitalized on promiscuous activity of a minimal set of enzymes [25,37], computational methods are currently in development with the aim to predict enzyme promiscuity at the genome scale level and to provide hints in the design of directed evolution experiments with practical application in white biotechnology (see Table 1 for an overview of the most relevant). There seems to be two distinct approaches to tackle these questions. The first approach takes into account enzyme sequence, structural features of proteins and reaction data such as substrate-products pairs. Carbonell & Foulon [38] developed a computational method that is based on molecular signatures as vector representations of protein sequences by its amino acids strings or *k*-mers, which are used to develop signature-based support vector machines (SVM). Mining was carried out on the non-redundant list of enzymes sequences in KEGG database [39]. From this tool, it was estimated that 28 ​% of the enzymes are catalytically promiscuous, whereas 40 ​% of them harbors substrate ambiguity. More interestingly, this computational analysis revealed that molecular signatures or *k*-mers in promiscuous enzymes were rather poor in aromatic and bulky amino acids whereas Gly and Ala were relatively more abundant. In addition, these signatures were found uniformly distributed in region of ≈20 ​Å around the catalytic site, which contrasted with non-promiscuous enzyme for which specific signatures were identified in this region surrounding the catalytic site. An interesting practical aspect of this predictor is to provide protein designers with a means of identifying the amino acid residues that are most critical for promiscuity and which can therefore be subjected to mutagenesis to optimize catalytic function towards one or other substrate. Another prediction tool termed ‘*Prom*iscuity *I*ndices *Es*timator’ or PROMISE was proposed by Chakraborty & Rao [40]. This predictor is based on method for active site detection which relies on the spatial and electrostatic properties of residues at the vicinity of the catalytic site of the proteins [41]. While enzymes with >39 ​% of charged and >20 ​% of polar residues in the vicinity (≈3 ​Å) of catalytic site have high probability to harbor promiscuous activity, this tool only considers catalytic promiscuity and has not been validated at the genome-scale level. To fulfil the limitation of these approaches, Xing et al. [42] developed Substrate-Product pair-based Enzyme Promiscuity Prediction (SPEPP) model, which is a deep-learning approach that incorporates substrate-product-enzyme triads gleaned from existing comprehensive reaction databases (*i.e.* KEGG [43], BRENDA [44]; MetaCyc [45], etc.). This method uses a deep neuronal network to elucidate the relationships between enzyme sequence features and substrate-product pairs and return a predictive score about the possibility of an enzyme catalyzing all potential substrate-product pairs. The model can address any substrate-product enzyme task in a high-throughput manner thus enabling to recognize novel enzymes with high potential to catalyze a reaction for a given substrate-product pair. It is noteworthy that the screen is limited to enzymes not exceeding 1000 AAs in length due to the constraints imposed by GPU memory limitations.

**Table 1:** Overview of computational methods and databases that can predict enzyme promiscuity, underground reactions, provides listing of compounds, reactions and enzymatic statistics.

Nickname	description	Web site access	Ref.
PROMISE	To assign a relative promiscuous index (PromIndex) to proteins with known active site and 3D structure. It is based on the previously described method for active site detection which relies on the spatial and electrostatic properties of the catalytic residues (CLASP)	no	[40]
PROXIMAL	Initially developed to predict xenobiotic metabolism and then further extended to predict promiscuity enzyme using reactant–product transformation patterns from the KEGG database	no	[53,55]
PROPER & GEM-PROPER	Genome-wide method to predict promiscuous functions of metabolic genes unsupervised manner. Based on a promiscuous ‘replacer’ function that may compensate for primary ‘target’ function of a gene that is either altered or lost. GEM-PROPER can predict cases of multicopy suppression in which functional promiscuous replacement happens ‘indirectly’ through a bypassing pathway or reaction	no	[47]
SPEPP	Substrate–product Pair-based Enzyme Promiscuity Prediction (SPEPP) model utilizes transfer learning and transformer architecture to predict enzyme promiscuity, thereby elucidating the intricate interplay between enzymes and substrate–product pairs. SPEPP allows users to incorporate candidate enzyme libraries from any source; this flexibility considerably broadens the scope of enzyme screening using *EnzyPick,* a web server platform	http://www.biosynther.com/enzypick/(no longer exist)	[42]
CLEAN	CLEAN (contrastive learning–enabled enzyme annotation) to assign EC numbers to enzymes with better accuracy, reliability, and sensitivity compared with the state-of-the-art tool BLASTp. CLEAN can (i) annotate understudied enzymes, (ii) correct mislabeled enzymes, and (iii) identify promiscuous enzymes with two or more EC number	no	[125]
MINE	MINE stands for Metabolic In silico Network Expansions (MINEs), is a metabolite database of computationally predicted enzymes promiscuity molecules for untargeted metabolomics that have not been observed, but that are likely to occur based on known metabolites and common biochemical reactions	https://minedatabase.mcs.anl.gov/#/home	[104]
cd-MINE	A comprehensive database containing spontaneous reactions that occur under physiological conditions and curated from literature as well as reactions predicted by generalized reaction rules	https://minedatabase.mcs.anl.gov/cdmine/#/home	[122]
MDFlow	Computational method to analyze/predict side effects of engineered modification on the microbial host metabolic network from enzyme promiscuity	https://github.com/HassounLab/MDFlow	[103]
ATLAS	Repository of known and predicted biochemical reactions between biological compounds listed in KEGG. It contains around 150,000 reactions, it used BrigIT as computational tool to assign known enzyme to novel and not known reaction	http://lcsb-databases.epfl.ch/atlas/	[57,58]

The other approach of predicting enzymatic promiscuity is based on promiscuous ‘replacer’ gene function, whereby the loss of function of a gene in a primary or classical metabolic route can be compensated either directly (the gene may encode an isozyme of the one encoded by the target gene) or indirectly (bypassing the metabolic block) by a promiscuous ‘replacer’ gene. A first initiative was brought about by Guzman et al. [46] who constructed a model-driven discovery of promiscuity from the dichotomy between *in silico* prediction of genes essentiality in the *E. coli* iJ01366 metabolic network model and failure of these genes to be essential experimentally. This type of failure argues on the existence of an alternative gene function that is missing in the model, and which can be searched for according to sequence homology using BLASTp at a high confidence score (<1E-40). This method enables the identification of a gene coding for a similar but secondary enzyme function (isozyme), whose overexpression will compensate for the loss of the target gene. In addition, only targets that lead to measurable phenotypic defect can be investigated for being compensated by an alternative promiscuous function. The tool termed the Enzyme Promiscuity Predictor or PROPER developed by Oberhardt et al. [47] goes a step further as it is able to predict all “target” - “replacer” genes pair by utilizing unsupervised PSI-BLAST based method for assessing potential secondary function of genes on a genome scale level in *E. coli.* Overall, PROPER could predict 2811 direct “target-replacer” gene pairs encompassing 794 metabolic target genes and 753 metabolic replacer genes. This tool was able to recapitulate previous known multicopy suppression works (see below), and 20 ​% of the predicted target-replacer genes pairs could be experimentally validated. These authors went one step further to develop GEM-PROPER that integrates PROPER with genome-scale metabolic models enabling to predict indirect promiscuous replacements, *i.e.* via metabolic bypasses. A total of 98 indirect target-replacer pairs were predicted by GEM-PROPER. Among them, the *pdxB* target whose loss of function abolishes the essential 1-deoxy-xylulose-5-P (DXP-dependent) pathway of pyridoxal-5′-phosphate synthesis has been predicted and validated to be replaced by *thiG* creating a DXP-independent pathway. In addition to these two distinct computational approaches, a pathway prediction algorithm termed GEM-Path developed by Campodonico et al. [48] enables to generate an atlas for commodity chemicals production routes in *E. coli*, which underlined novel reactions that need novel -very likely-promiscuous enzymes. On the same line, pathways search for a given compound through retrobiosynthesis can also highlight the need of a promiscuous enzyme to catalyze a particular reaction and this identification is obtained by machine learning that exploit enzymes sequences and chemoinformatic representation of chemical transformations [49,50].

Altogether, these various computational approaches are very compelling to identify enzymes with promiscuous activity, which notably has enabled to expand the native genome-scale metabolic network such as *E. coli* K12 iJO1366 [51] with new so-called underground reactions as revealed by identified promiscuous enzymes with PROPER [52]. In a similar way, Amin et al. [53] created an extended metabolic model (EMM) of the iML1515 *E. coli* model system [54] by implementing promiscuous reactions predicted from the PROXIMAL tool [55]. At variance to PROPER, PROXIMAL uses information on the chemical neighborhood (atom types of two-level nearest neighbors) around the reaction center to predict transformation of a given compound into a list of putative products and their cognate reactions catalyzed by promiscuous activity of enzymes present in the host system. The list of products can be confounded with the *E coli* metabolic database (ECMD, [56]), giving support to the prediction. With this computational workflow, the iML1515 metabolic model was augmented by 23 new reactions and 16 novel metabolites. Apart from these prediction tools, there are some resources such as ATLAS [57,58], which is a repository of 130,000 hypothetical enzymatic transformations computed by BNICE algorithm (Biochemical Network Integrated Computational Explorer; [59]. This algorithm employs computational chemistry methodologies to generate every possible reaction for a given set of chemical reaction rules and starting compounds and the MINEs database [60], which compiles a list of 571,000 compounds predicted by BNICE. However, in the near future, it is expected that machine learning methods, fed by validated experimental data, and combined with proven algorithmic methods for protein structure analysis will lead to understand what confers promiscuity to an enzyme, which would be instrumental to better improve enzymes and pathways evolution.

## 3 Enzyme promiscuity as a cornerstone in synthetic biology

Inspired from natural evolution, enzyme promiscuity is the starting place to create a new metabolic function with the ultimate aim of making the catalyst as specific and effective as possible on the new often non-natural- substrate and eventually, but not obligatorily, losing its activity on its primordial one. The construction of the SACA pathway, which stands for synthetic acetyl-coA pathway is an inspiring example of the use of enzyme promiscuity to create a synthetic pathway from capturing C1-carbon (formaldehyde) into acetyl-CoA in only 3 steps. The first step requires the condensation of two molecules of formaldehyde into glycoaldehyde, which is carried out by an engineered benzoylformate decarboxylase (BFD, EC. 4.1.1.7). The BFD belongs to the family of lyases, specifically the carboxy-lyases, which decarboxylates benzoylformate into benzene and CO_2._ This cleavage of carbon-carbon bonds requires a thiamine diphosphate as cofactor which otherwise can harbor another intrinsic property of activating an aldehyde to form a dimer with another aldehyde. A very weak activity of formaldehyde condensation into glycoaldehyde was detected with BFD of different origin (k_cat_/K_M_ ​≈ ​0.1 ​M^−1^ ​s^−1^), and this activity was increased about 100-fold in a variant bearing 7 mutations, with five of them located in the active center. The next step was to repurpose the promiscuous phosphoketolase (PKT, EC:4.1.2.9/4.1.2. 22) enzyme into an acetyl-phosphate synthase (ACPS), taking into account that the intermediate in the catalytic mechanism of PKT is the 2 α,β-dihydroxyethylidene-ThDP, which is the key intermediate to form acetyl-P (AcP) from F6P or X5P as natural substrate. Quite interestingly, glycoaldehyde can interact with ThDP to form this intermediate, thus potentially yielding AcP. From 8 PKT screened from various bacterial sources, 5 of them were able to produce AcP when incubated with glycoaldehyde and Pi with the best one exhibiting k_cat_/K_M_ 3.2 ​M^−1^ ​s^−1^. The last step is catalyzed by the natural phosphate acetyltransferase (PTA). When assembled *in vitro*, the SACA pathway yielded stoichiometry amount of AcP from formaldehyde. However, the *in vivo* functioning of the pathway was still very limited because of very weak catalytic capacity together with inhibition of ACPS by formaldehyde, while exhibiting advantages over other methanol/formaldehyde pathways of being carbon-conservative, ATP-independent and be operational under aerobic and anaerobic conditions. The promiscuous activities of the generalist phosphoketolase enzyme has been also successfully exploited to create carbon-conservative cycle pathways enable to achieve 100 ​% yield of acetyl-CoA from any monosaccharides in both yeast and bacteria [[61], [62], [63]].

The construction of a non-natural pathway by mimicry with a natural pathway presenting substrates of similar structure to those of the synthetic pathway is another example that illustrates the critical role of enzymatic promiscuity. This strategy has been applied for the synthesis of the non-natural chemical synthon 2,4-dihydroxybutyric acid (DHB) by mimicking the aspartate -homoserine pathway [64]. The first reaction involved the phosphorylation of malate into malyl-P. L-Malate is a C4 dicarboxylic acid that differs from L-aspartate by the presence of a hydroxyl group at carbon C2 instead of an amine (NH_2_) function. Although the *E. coli* aspartate kinase LysC (EC 2.7.2.4), which catalyzes the phosphorylation of aspartate into aspartyl-P does not show any measurable activity on L-malate, it was originally reported that L-malate competitively inhibited this reaction [65], arguing that this metabolite has access to the active site. Therefore, through rational mutagenesis, LysC was converted into a malate kinase, which required two mutations in the catalytic site (V115A, E119S) and a third mutation (E434K) located at the enzyme surface. The variant LysC ^V115A E119S E434K^ resulted in a complete loss of activity on aspartate while the activity on (L) malate was approximately twofold lower than that of the wild-type enzyme acting on aspartate [64]. The next step which converts malyl-P into malate-semialdehyde was catalyzed by a mutated aspartate semialdehyde dehydrogenase from *B. subtilis*, whereas the last step that reduces malate semialdehyde into DHB was catalyzed by a bacterial enzyme annotated as 4-hydroxybutyraldehyde dehydrogenase. Another prominent example of substrate promiscuity is the polyhydroxyalkanoate (PHA) synthase (EC.2.3.1. 304) that was engineered to be able to make polylactic polymer using D-lactyl-COA while the natural activity of this enzyme is to build polymers from 3-hydroxyalkanoate of 4–12 carbons length or 4-hydroxybutanoate but not 2-hydroxyalkanoate. The mutant PHAC1 STQK, with the double mutation S325T and Q481K was fully functional of lactyl-COA, while maintaining its original reactivity on conventional monomer substrates [66]. Overall, novel enzymes in synthetic non-natural pathways can be constructed starting from promiscuous activity, even if the catalytic efficiency of these enzymes was >10^4^-fold lower than their ‘physiological’ or natural substrate. It's worth noting that engineering promiscuity can also be relevant to industrial applications, as has been demonstrated to expand the substrate panel of chloramphenicol acetyl transferase (CAT) to use it as an alcohol acyl transferase (AAT) platform for the synthesis of different esters from various alcohols and acyl CoA [67]. These authors found that mutation of Tyr a position 20 to phenylalanine weakens the interaction between this aa residue, chloramphenicol and the catalytic H189 at the transition state giving rise to a promiscuous CAT able to accept at least 21 alcohol and 8 acyl-CoA substrates for microbial biosynthesis of linear, branched, saturated, unsaturated and/or aromatic esters. This works demonstrates that promiscuity can be exploited either way, and in this case, repurposing CATs rather than bioprospecting various AATs is a relevant approach for production of various esters by biocatalysis or bioconversion that have broad applications as flavors, fragrance, fuels and solvents [68].

## 4 Enzyme promiscuity for emergence of hidden pathways with potential biotechnological relevance

The pertinent case studies described above illustrate our ability to create new, unnatural metabolic pathways almost from scratch, using as a starting point an enzyme with very low, or even with no activity at all on a non-natural substrate, which therefore requires extensive protein engineering to yield suitable activity. These extreme cases rather show how much an enzyme can evolved and acquires novel catalytic activity. However, many more enzymes than anticipated display side activity, giving rise to the emergency of hidden metabolic pathways that constitute what has been termed “the underground metabolism” [69]. This underground metabolism encompasses both metabolic routes that are leaks in the normal metabolic network and which are in most case detrimental for the physiology of the cell and serendipitous pathways (SPs), which according to Copley and colleagues [70] can reach by mutations or evolution metabolic flux that is physiological relevant so as the enzymes in these SPs are no longer promiscuous.

Serendipitous pathways are metabolic routes that can complement or bypass a primordial pathway that has been interrupted by the loss of an enzyme. As described above, PROPER was developed to identify *in silico* potential promiscuous replacer genes that can provide this solution, while the experimental validation requires specific technique among which the multicopy suppression method is the most appropriate. This strategy which has been established long ago [71] requires the availability of a gene library in a multicopy plasmid such as the *ASKA* library, which contains every *E. coli* ORF individually cloned into a high copy plasmid under T7 inducible promoter [72] or a high-copy yeast *S. cerevisiae* genes library [73]. Suppression of the defective phenotype resulting from deletion of a gene encoding a critical function is obtained by transforming the mutant with these libraries. Hence, in addition of recovering the wild-type gene among the suppressors, it is expected to find out suppressor genes that very likely encode isozymes or enzymes with promiscuous activity, since their overproduction would have generated sufficient activity to replace the native enzyme. Patrick et al. [74] addressed the prevalence of enzyme promiscuity within the *E. coli* proteome by transforming 305 ​*E. coli* mutants from the single-gene knockout strains collection [75] unable to form colonies on a simple M9-glucose medium with the ASKA library. This experiment was very informative showing that >20 ​% of the mutants were rescued by overexpression of suppressor genes encoding enzymes with catalytic promiscuity or substrate ambiguity different from the native ones, even though many of them still remain to be enzymatically characterized. Also, many of these suppressors encoded enzymes able to generate a metabolic bypass. Equivalent genome-wide study has not been carried out yet with the yeast KO collections (http://www.euroscarf.de/index.php?name&equals;Description) transformed with a high copy library to distinguish whether gene paralogs, which is more prominent in yeast than in *E*. *coli* due to genes duplication, would arise more frequently as suppressor than genes encoding promiscuous enzymes.

**Fig. 3:**
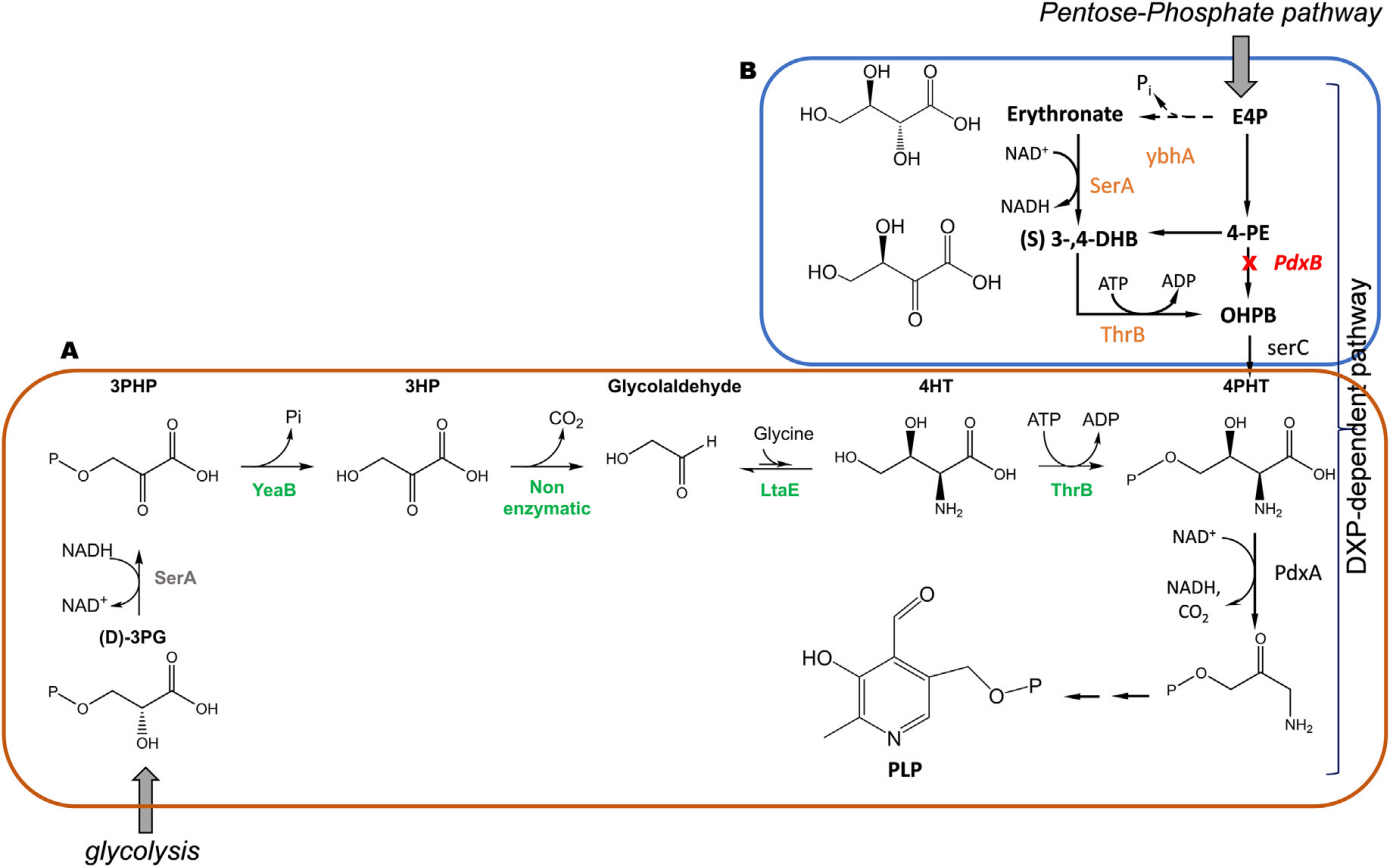
Scheme of the underground pathway to overcome pyridoxal-5-P growth-dependency in *E. coli* as revealed by multicopy suppression strategy (A) and by evolutionary engineering (B). Abbreviation: DXP: deoxyxylulose -5P; E4P: erythrone-4-P; 4-PE: 4-phosphoerythronate; OHPB: 3-phospho-4-hydroxy-phospho-a-ketobutyrate; 4PHT: 4-phophohydroxy-L-threonine; PLP: pyridoxal-5′-phosphate; D-3PG: D-3-phosphoglycerate; 3PHP: 3-phosphohydroxypyruvate; 3HP: 3-hydroxypyruvate; 4HT: 4-hydroxy-L-threonine; YeaB; putative 3-phosphohydroxypyruvate phosphatase activity; LtaE: L-threonine aldolase; ThrB: homoserine kinase; SerA: phosphoglycerate dehydrogenase; SerC: 3-phosphorserine amino transferase; PdxB: 4-phophoerthryonate dehydrogenase, PdxA: 4-pphoshydroxy-L-threonine dehydrogenase. See further details in the text.

Multicopy suppression was also employed to highlight serendipitous pathways in *E. coli* able to bypass the *pdxB*-dependent pathway that produces pyridoxal 5′-phosphate (PLP). PLP is required for the function of at least 60 enzymes in the *E. coli* proteome and therefore this cofactor is essential for growth. Using ASKA collection, Kim et al. [76] isolated seven different genes whose overexpression could restore growth a *pdxB* mutant. Surprisingly, they did not find any alternative enzyme that was able to replace the 4-phosphoerythronate dehydrogenase (4-PE) encoded by *pdxB*. They rather identified three serendipitous pathways that were totally outside the DXP-dependent pathway [77], and which diverted intermediates of natural pathways to convert them into intermediates downstream of the PdxB-catalyzed step. Till now, only one of the three serendipitous pathways has been fully characterized (Fig. 3). It connects the glycolytic intermediate 3-phosphoglycerate (3-PG) which also a precursor of serine to PLP through 5 reaction steps, including the homoserine kinase encoded by *thrB*, which exhibits promiscuous activity on L-4-hydroxythreonine to form L-4-phosphohydroxythreonine as immediate precursor of PLP, the low-affinity threonine aldolase encoded by *ltaE* that catalyzes the condensation of glycine and glycoaldehyde into 4-hydroxythreonine, a broad specific phosphatase encoded by *yeaB/nudL* that dephosphorylates 3-phosphohydroxypyruvate into 3-hydroxypyruvate, which is thereafter non enzymatically decarboxylated into glycoaldehyde and finally the phosphoglycerate dehydrogenase encoded by *serA* that oxidizes 3-phosphoglycerate obtained from serine into 3-phosphohydroxypyruvate [76]. A fourth metabolic bypass able to rescue growth of *pdxB* mutant was disclosed from the Enzyme Promiscuity Predictor PROPER (see above for its description). This tool predicted that overexpression of *thiG* encoding 1-deoxy-D-xylulose 5-phosphate: thiol sulfur transferases could rescue growth defect of a *pdxB* mutant on M9-glucose, which was validated experimentally [47]. The protein ThiG shows similarity to the glutamine amidotransferase component of the pyridoxal 5′-phosphate synthase complex encoded by *pdxS* in *Bacillus subtilis* and structure model using the *Bacillus* enzyme revealed that ThiG protein shares with PdxB same fold and overlapping residues in the active sites [47]. Of note, this gene was not found within the multicopy suppressor of *pdxB* mutant by Kim et al. [76], which indicates that this multicopy suppression method has some limitation as for instance insufficient expression of the gene. Overall, and as stressed by Rosenberg & Commichau [78], high throughput suppressor screen is a power method to identify enzymes with promiscuous activity. In addition, the identified enzymes could be further improved by directed evolution to optimize these novel pathways. However, this strategy relies on easy scoring phenotypes such as recovering of growth defects (*i.e.* auxotrophy for an essential factor) or growth on a non-natural substrate, and therefore functional profiling based on non-targeted metabolomics [79] or on droplet-based microfluidic coupled to enzyme assay [80] have been set up to partially overcome these limitations. Also, this approach may prove unsuccessful in the event that overproduction of proteins or accumulation of intermediates are toxic for the cells.

**Fig. 4:**
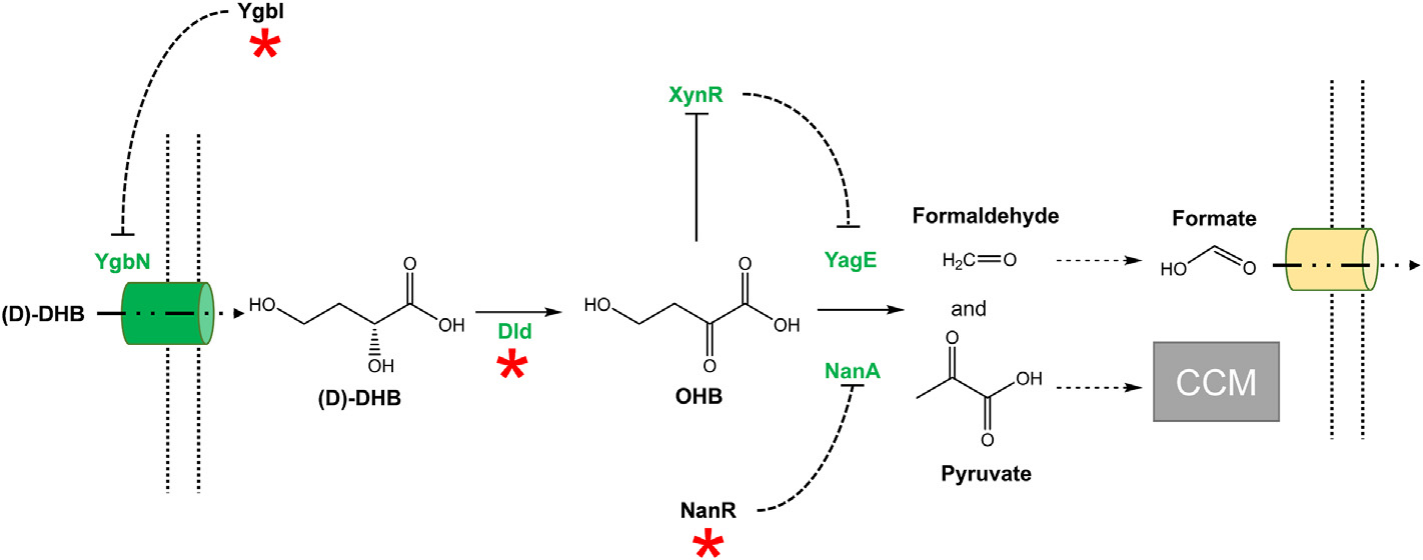
Scheme of the underground pathways implicated in DHB assimilation in *E. coli* as revealed by combining transcriptomic and evolutionary engineering approaches. Red Asterix indicates genes that were mutated to yield protein variant necessary to enable growth of *E. coli* on DHB. Abbreviations: DHB: (D)- 2, 4-dihydroxybutyrate; OHB: 2-oxo-4-hydroxybutyrate; CCM: carbon central metabolism. See text for additional details.

The multiple copy suppression approach is not appropriate to underline underground reactions which upon subtle and often gradual mutational events could yield metabolic novelties. The adaptive laboratory evolution (ALE) method, applied to relatively fast-growing microorganisms such as *E. coli* and yeast *S. cerevisiae* is well suited as it can address evolution strategy in a relatively short time experiments [17,[81], [82], [83]]. In addition, the way in which ALE experiments are carried out may help to address relevant questions related to evolutionary mechanisms, namely whether the early mutational events of adaptation specifically target enzymes with promiscuous activities, and whether these promiscuous enzymes continue to evolve in order to select the most fitness or efficient cells in relation to the constraint initially imposed. These questions have been addressed by Guzman et al. [46,84] in their quest to experimentally validate the computational prediction of underground enzymatic reactions and pathways enabling growth of *E. coli* on a large set of non-natural carbon substrates, including D-lyxose, D-tartrate, D-arabinose and ethylene glycol. They have divided the ALE experiments in two phases. A first phase, called the “weaning phase”, corresponds to the dynamic stage during which cells acquire metabolic novelties which are either the capacity to growth on an exotic carbon source or to repair a metabolic disfunction caused by a mutation. This phase is followed by a second “static phase”, during which strong selection pressure is exerted to select the cells with the best fitness for these new growth conditions. ALE experiments are generally characterized by “jumps” in growth rate caused by a causal mutation that is fixed in the cell population and genetically stable [85]. In regard to the first question, independent studies looking for non-native substrates assimilatory pathways [84,86] or for repairing metabolic defect causing growth defect such as inability to synthetize pyridoxal 5′-phosphate [87] or coenzyme A [88], key mutations obtained at the early stage of adaptation fall into two categories. The first category was represented by mutations in regulatory genes, *i.e* genes encoding transcription factors or mutations in intergenic regions that likely affects sigma factor binding and transcription of target genes needed for these adaptations. Major effects of these regulatory mutations are the increase in expression of genes encoding enzymes with promiscuous activities and thus an increase in the dose of low-level side activity of these enzymes. The other category concerns structural mutations, *i.e.* mutations in genes encoding a promiscuity enzyme whose catalytic efficiency on the non-natural substrate is strongly increased. Moreover, these studies have shown that combination of regulatory and structural mutations is often required for adaptation, such as the growth of a *ΔpanD* mutant that is unable to synthetize β-alanine as precursor for the essential cofactor coenzyme A, was rescued by the combination of *rutR* deletion, leading to upregulation of the uracil degradation pathway into malonic semialdehyde (MSA), overexpression of the promiscuous GabT transaminase that converts MSA into β-alanine and loss of function of Upp that catalyzes the formation of UMP from uracil [88]. Malfoy et al. [89] also showed that the capability of *E. coli* to grow on the non-natural chemical synthon 2, 4-dihydroxybutyric acid (DHB) required the combination of three genetic modifications, which included the loss of function of *ygbJ* which encodes a transcription repressor of the threonate/erythronate operon [90] resulting in the depression of *ygbN* encoding a putative D-DHB transporter, a point mutation in the promoter region of *dldD* encoding a promiscuous D-lactate dehydrogenase resulting in a 15-fold increase in the activity on D-DHB and deletion of *nanR* that encodes a transcription repressor of sialic acid pathway genes [91], leading to ​> ​100 fold increase in the expression of *nanA* coding for the promiscuous *N*-acetyl neuraminic acid lyase. In the same time, the OHB which is formed by oxidation od DHB could trigger expression of *yagE* encoding a pyruvate-formate lyase due to its capability to inhibit the transcription factor XynR that represses the D-xylonate dependent *yagGEF* operon ([92], Th Malfoy & J François, unpublished data). However, quite surprisingly, the loss of function of *nanA* but not that of *yagE* abrogated the growth of *E. coli* on DHB while *in vitro* only YagE showed measurable activity of cleavage of OHB [89]. Thus, the huge transcriptional increase nanA was necessary to account for the *in vivo* cleavage OHB into pyruvate and formaldehyde (Fig. 4). At variance, a single mutation in the structural gene *yihS* encoding a promiscuous isomerase activity [93] or *dlmA* encoding the promiscuous D-malate dehydrogenase enzyme [30] was sufficient for growth on D-lyxose and m-tartrate, respectively [84]. Likewise, mutations in *serA* encoding 3-phosphoglycerate dehydrogenase were also found to rescue growth of *pdxB* mutant on minimal glucose medium, resulting in a bypass pathway involving erythronate, which is oxidized into 2,3-dihydroxy-oxobutanoate by the mutated SerA followed transamination catalyzed by the promiscuous SerC into 4-hydroxythreonine [87] (Fig. 3B). In all cases, the mutation leads to an increase of the catalytic efficiency (k_cat_/K_M_) of the enzyme on the non-natural substrate. In the static phase of ALE experiments, numerous new mutations accumulate, many of which target promiscuous enzymes already identified in the weaning phase, leading to a further increase in their catalytic efficiency on the non-native substrate. However, other genes encoding additional promiscuous enzymes or regulatory factors are targeted, leading overall to optimized growth to eventually get best fitness cells towards the new environmental condition. Of note, Pontrelli et al. [88] used another ALE strategy to evaluate how flexible can be the metabolic pathways to adapt to extreme metabolic constraints. To this end, they used a *ΔpanD* mutant that is unable to produce β-alanine, a key precursor of the coenzyme A. An evolved PS1 strain able to grow on M9-glucose without complementation was isolated, revealing that CoASH was obtained through redirecting the uracil degradation pathway into malonyl-semialdehyde (MSA) which was converted into β-alanine by the promiscuous GabT transaminase. They then deleted this bypass pathway, subjected the strain to a second ALE and isolated an evolved strain, which harbored a gain-of-function mutation on ornithine decarboxylase (SpeC) displaying both oxidative decarboxylation and deamination reaction. Having eliminated this second pathway, the authors identified a third pathway after ALE, which involves the degradation of polyamines using enzymatic reactions that have yet to be characterized.

Overall, these studies highlight the crucial role of enzyme promiscuity in evolutionary adaptations. They underline the flexibility of the cell's metabolic network, when subjected to strong constraints on growth and viability and identify the rich reservoir of underground metabolic pathways that could be exploited for biotechnological purposes. Along this line, Kovacs et al. [52] argue that underground metabolism can enhance the production yield of industrially relevant compounds and even enable the production of new compounds that cannot be produced by native metabolic pathways. Their assumption was based on the reconstruction of a model of the metabolic network of *E. coli* (model E.coli MG1655 iJO1366 developped by Ref. [51]) by integrating both experimentally validated [36] and predicted underground reactions by PROPER algorithm [47]. This new reconstructed and expanded model with underground metabolism proved to be valuable in confirming that the aldehyde reductase encoded by *gldA* which has a primary activity on reducing dihydroxyacetone into glycerol is also capable to convert methylglyoxal into lactaldehyde as secondary activity and this reaction is necessary to enhance the production of 1,2-propanediol [94]. More interestingly, the model enables to identify two underground metabolic reactions for 1-propanol synthesis that involve the acetaldehyde dehydrogenase (acetylating) enzyme encoded by *mhpF,* which produces propionyl-aldehyde from propionyl-CoA and the broad specific NADPH-dependent aldehyde reductase encode by *yqhD,* which reduces propionyl aldehyde into 1-propanol. However, these computational tools that utilize enzymes promiscuity to find alternative pathways for production of biotechnological relevant compounds are distorted by two singularities. On the one hand, simple flux balance analysis (FBA) is used without any thermodynamic constraints and consequently many of the predicted pathways may be unrealistic under physiological conditions. On the other hand, these tools can predict neither potential damage caused by activating underground reactions nor potential interference with native metabolism due for instance by accumulation of toxic intermediates, as we will develop further below.

## 5 Enzymatic promiscuity penalizing pathway engineering for bioproduction

As much as enzymatic promiscuity can be beneficial in the engineering of bioproduction pathways, it can also be detrimental to these same designs. The disruptive action of enzymatic promiscuity may first manifest itself in the siphoning off of one or more intermediates in the bioproduction pathway, thereby reducing production titre and yield (Fig. 3). A relevant example is the negative impact of endogenous promiscuous phosphatases on the phosphorylated intermediates in the terpenoid biosynthesis pathway. Many intermediates in this pathway are phosphorylated and are prone to dephosphorylation by the widespread phosphatases expressed in the *E. coli* proteome [95]. Wang et al. [96] reported genetic evidence that 28 out of 56 phosphatases that were retained based on high expression in the transition of exponential to stationary phase of growth had a negative impact on lycopene production and suggested these enzymes should be novel metabolic engineering targets to further enhance the titer of many important chemicals derived from terpenoid pathway. Another example deals with the interference of promiscuous pyruvate-dependent aldolytic cleavage of 2-oxo-4-hydroxbutyrate (OHB) in the bioproduction of the non-natural compound 2,4-dihydroxybutyrate (DHB). The effect of these aldolases, encoded by at least seven different genes [[97], [98], [99]], cleaves OHB into pyruvate and formaldehyde [86], causing siphoning of this intermediate, reducing DHB production titer accompanied by potential toxicity due to formaldehyde production (C. Alkim & J François, unpublished). The recent understanding of the lysine degradation pathway in *E. coli* involving a promiscuous transaminase and dehydrogenase [100] is another example, although whether this metabolic perturbation results in reduced lysine production remains to be shown. The competition between different substrates is another possible source of metabolic disruption caused by enzymes with promiscuous activity. As an example, is the finding that xylose reductase and xylitol dehydrogenase can also act on galactose and galactitol, resulting in accumulation of galactitol and tagatose which can impair sugars fermentation [101]. Competition of the promiscuous activity of phosphohomoserine kinase encoded by *thrB* to phosphorylate various non-natural intermediates including L-4-hydroxythreonine, 3,4-dihydroxy-2-oxobutanoate and hydroxypyruvate can lead to deleterious effect of threonine synthesis from L-homoserine which requires its ThrB-dependent phosphorylation [87,102].

**Fig. 5:**
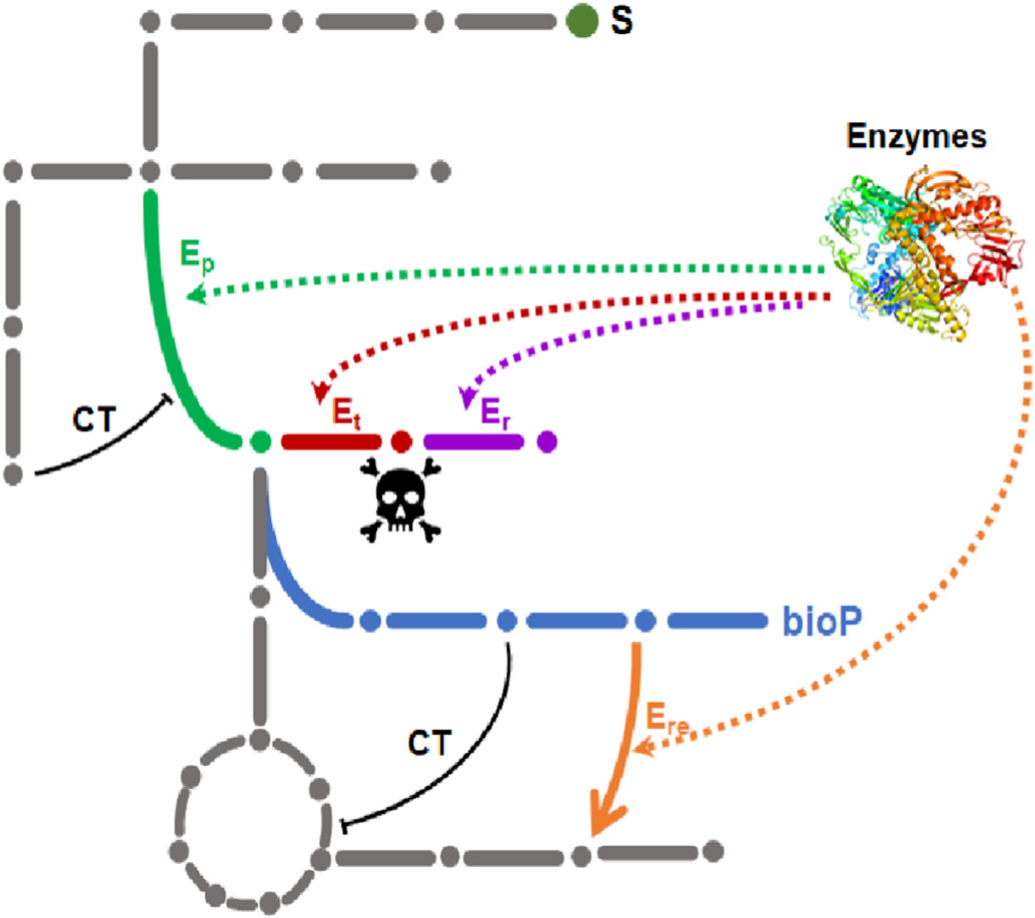
Schematic design representing the possible effects of hypothetical promiscuous enzymes in the metabolic network of a microbial host cell. **Grey lines** schematically represent the native metabolic network, **blue lines** illustrate a heterologous/non-natural pathway yielding a bioproduct from S1 (BioP). The **green arrow** identifies an underground reaction catalyzed by a promiscuous enzyme Ep; the **red arrow** identifies a metabolite damage reaction leading to a toxic compound illustrated by a deadhead and catalyzed by E_t_. This toxic compound can be degraded by the repair enzyme Er indicated **in purple**. The **orange arrow** represents a reaction that is catalyzed by a promiscuous enzyme E_re_ which can reroute an intermediate of the non-native pathway to the native metabolic network, resulting in a reduced flux in the production pathway to yield BioP. Cross-talks (CT) between native and non-natural metabolic pathways are illustrated by **black bars**, and are mediated by metabolite intermediates, either natural (**grey** dot) or non-natural (blue dot).

Metabolic interference through inhibitory crosstalk constitutes a third level of negative effects on the efficiency of bioproduction pathways (Fig. 5). The seminal example of such inhibitory cross-talk has been illustrated by the activation of an underground pathway for the synthesis of pyridoxal-5′-phosphate that bypasses the native and essential PdxB-dependent pathway ([102] and **s**ee Fig. 3). Two intermediates in this novel pathway, namely 3-hydroxypropionate (3HP) and 4-hydroxythreonine (4HT) are not normal metabolite in *E. coli*. 3HP was found to interfere in the synthesis of serine and branched amino acids according to three different mechanisms. 3HP competitively inhibits threonine deaminase encoded by *ilvA*. It also acts as a suicide inactivator of one the acetohydroxyacid synthase encoded by *ilvB*/*ilvN*. The third mechanism is the condensation of 3HP with pyruvate catalyzed by the other acetohydroxyacid synthase encoded by *ilvH/M* into a dead-end product 2-aceto-2,3-dihydroxypropanoate (ADHP). The toxic action of 4HT is due to its competition with homoserine for homoserine kinase encoded by *thrB*, as both substrate are phosphorylated by this enzyme, and hence high level of 4-HT compared to homoserine can be lead to threonine auxotrophy.

Given the above consideration, metabolic disruptions have been until now largely identified from experimental studies arising from unexpected phenotypic behaviors of the engineered microbial host. Predicting these perturbations early in the design cycle would be highly beneficial not solely by reducing experimental efforts but in improving production performance of the microbial cell factory. In this regard, Porokhin et al. [103] developed a computational method termed ‘Metabolic Disruption Worflow’ (*MDFlow*) to discover interactions and metabolic network disruption arising from enzyme-substrate promiscuity (Table 1). This method utilizes PROXIMAL to reconstruct reactions from enzymes promiscuity (see section 2 and [55] and relies on FBA to assess the impact on product yield, biomass and growth rate of the host before and after pathway design. This tool has been designed to manage interference on the metabolic network in one of two following scenarios. Scenario 1 predicts metabolic network interference due to overexpression of a promiscuous enzyme, either native or heterologous, with activity on natural host metabolites. In scenario 2, prediction is made considering that a native enzyme with promiscuous activity will interfere with new (non-natural) intermediate(s) of the new (non-natural) pathway implemented in the host cell. The authors tested *MDFlow* with the experimental data reported by Patrick et al. [74], who looked for suppressor genes whose overexpression could rescue the growth of essential genes on M9-glucose. This case study corresponds to scenario 1. Due to the constraints of FBA, which could only reproduce growth arrest for 15 genes deletions out of 41 experimentally observed using the iML1428 metabolic model of *E. coli*, the *MDFlow* model was able to find for 9 of them the same suppressor genes as that of Patrick et al. work. This computational tool was also used to predict metabolic interference causing the reduction of 3-hydroxypropionic acid yield from expression of three different heterologous pathways for production. They showed that the loss of 3HP yield was higher in scenario 2 than in scenario 1, since the more promiscuous enzymes involved in the heterologous pathway, the greater the interference with the native and heterologous pathways. Overall, *MDFlow* is the first systematic automated tool enabling to predict metabolic network disruption in engineered microbial cells. This computational tool can be further enhanced using *e.g* MINE, which can increase the number of predicted enzyme promiscuity reactions and products [104]. In addition, better prediction will anyway require additional biological data such as concentration of metabolites and enzymes activity in non-engineered and engineered cells.

## 6 Enzyme promiscuity causing metabolite damage and importance of repairing enzymes

Metabolic damage caused by promiscuous enzyme activity is probably the most delicate metabolic disturbance to detect and has long been under-diagnosed, probably because cells are endowed with damage control systems that can be considered equivalent to their DNA and protein macromolecule repair systems [105,106]. The difficulty in identifying such metabolic damage is largely due to the fact that it occurs in very specific situations, such as when a non-natural pathway is implemented in a microbial cell for bioproduction, and the more alien the pathway, the higher the risk of metabolic damage is to the cell, or in metabolic diseases in human, such as L-2-hydroxyglutaric aciduria, which is due to a defect in the repair enzyme L-2-hydroxyglutarate dehydrogenase, leading to a toxic accumulation of L-2-hydroxyglutarate in tissues [107]. Metabolic damage has two origins: spontaneous chemical reactions taking place *in vivo*, or side activity of enzymes with promiscuous activity. However, damage products whether spontaneous or resulting from enzymatic reactions, are useless or toxic and can inhibit essential metabolic enzymes. Therefore, they can seriously compromise the growth and physiology of the living system. To repair these damages, the cell has invented at least two types of damage control systems: the first one relies on metabolite repair enzyme(s) which among other things, can revert the damage product into a physiological intermediate whereas the other system prevents metabolite damage using preemption enzymes that can either destroy or neutralize reactive intermediates [60,105,106]. There is no place in this opinion paper to report on several examples of metabolite damage and repair systems, as they have been nicely covered in excellent review papers [60,105,108,109]. However, it is worth to comment on the paradoxical case of a repair enzyme which has a conserved phosphatase activity among bacteria, yeast or mammals. In mammals, this enzyme, known as phosphoglycolate phosphatase (PGP), dephosphorylates both 4-phosphoerythronate and 2-phospholactate [110]. These two molecules are toxic products illicitly formed by the secondary action of glyceraldehyde dehydrogenase and pyruvate kinase, respectively. The accumulation of the former leads to a reduced flux in the pentose phosphate pathway through inhibition of 6-phosphogluconate dehydrogenase, whereas the accumulation of the later causes a slowdown of glycolysis through impaired formation of its main activator, fructose-2,6-P_2_ via PFK2 inhibition [111]. In yeast, the orthologue of PGP is encoded by *PHO13*. There have been numerous reports showing that ablation of *PHO13* in yeast improves ethanol production from xylose without clear explanation of this effect [[112], [113], [114], [115], [116], [117]]. A tentative explanation of this effect was proposed by Bommer et al. [118] based on the fact that 4-phosphoerythronate accumulates in a *pho13Δ* mutant, causing inhibition of 6-phosphogluconate dehydrogenase. This inhibition enables to adjust flux rate of the NADPH-dependent xylose reductase to that of the NADH-dependent xylitol dehydrogenase, avoiding toxic accumulation of xylitol and depletion of NADPH. However, this explanation is probably not complete, as it has been found that the loss of function of *PHO13* also stimulates ethanol production from xylose in engineered strains expressing the xylose isomerase system [117]. A phosphatase acting in a similar manner as the human PGP and yeast Pho13p has been recently discovered in *Bacillus subtilis*. However, this enzyme encoded by *cpgA* carries another activity that is implicated in ribosome assembly, cell morphology and cell wall synthesis [119,120]. CpgA is thus ascribed as a moonlighting protein. Nevertheless, a *ΔcpgA* mutant of *B. subtilis* is unable to grow on glucose or gluconate, whereas it grows well on malate, unless glucose is also present. This growth defect has now been ascribed to an inhibition cascade of pentose phosphate pathway and glycolysis triggered by 4-phosphoerythronate. This toxic molecule produced from erythrose-4-P by the illegitimate action of GAPDH, accumulates due to the lack of CpgA phosphatase activity. It then causes an inhibition of 6-phosphogluconate dehydrogenase, leading to accumulation of 6-phosphogluconate, which in turns inhibits the glycolytic phosphoglucose isomerase. Support to this mechanism came from the overexpression of the yeast *PHO1*3 that enabled recovering growth of a *ΔcpgA* mutant on glucose and that CpgA displays a broad phosphatase activity with 4-phosphoerytronate as its preferred substrate [121].

As already mentioned above, metabolite damage can also arise from spontaneous chemical reactions. These events are often neglected whereas metabolites are in fact not passive objects but several of them can react spontaneously and this spontaneous reaction rate can be exacerbated in microbial host expressing heterologous or non-natural metabolic pathways for bioproduction. To predict spontaneous metabolic reactions that can happen under these circumstances, Jeffryes et al. [122] have created the Chemical Damage (CD) -MINE resource (Table 1). This database is a compendium of curated and predicted metabolic reactions that can occur spontaneously under physiological conditions. It integrates a set of 148 manually curated chemical reaction rules that target the ModelSEED database [123] comprising 24,087 compounds with defined chemical structure leading to 165,720 compounds obtained from spontaneous reactions. Thus, CD-MINE predicts spontaneous reaction based on compounds structure and reactivity. The authors report the case studies of anthranilate production by *E. coli*, as this compound is oxygen sensitive and highly reactive with other metabolites. The CD-MINE predicted 13 derivatives from spontaneous reactions of anthranilate among which acetylated or methylated form of anthranilate as well as derivatives of benzoate that can form radicals in the presence of hydroxyl radicals. All of these compounds could be eventually prevented by expressing catalase and *N*-acetyl anthranilate amidase to avoid deleterious effects of these toxics on anthranilate pathway production. Of course, enzymatic catalysis has the advantage to accelerate the reaction rate and therefore to overpass spontaneous reaction. However, reaction rules in CD-MINE relates to kinetics of the reaction and size of the metabolites pools that the reaction may operate in physiological condition. CD-MINE is accessible through a dedicated website that is very useful to query data either by textual information or metabolic pathways (https://minedatabase.mcs.anl.gov/cdmine/#/home).

## 7 Concluding remarks and perspectives

A shift from our linear -petrol-based- economy to circular -renewable carbon-based- economy is strongly awaited. It will rely in part on our ability to engineer microbial cells able to produce at high titer, yield and rate various drop-in chemicals that will be platform molecules able to advantageously replace some of those obtained from fossil resources. Achieving this goal will be a long journey because we are far from being able to master any biological systems in comparison to our ability to build highly performant computers. Among the many challenges to be overcome is the fact that more enzymes than anticipated have promiscuous activities that can be exploited wisely for bioproduction, but meanwhile they can cause interference between natural and synthetic metabolisms, causing deleterious effects on growth and production. In addition, enzyme promiscuity is at the onset of a rich reservoir of underground reactions that can be either beneficial for biotechnological purposes, or counterproductive, as it can be at the origin of metabolite damages. The finding that the overexpression of >200 genes with largely unknown function was found to positively affect carotene production in yeast [124] indicated that our understanding of underground reactions mediated by enzyme promiscuity is still very rudimentary. Also, metabolites that can be subject to spontaneous -non-enzymatic- reaction should not be neglected notably in cell expressing heterologous/non-native metabolic pathways. So far, all the information gathered on enzyme promiscuity, metabolic network interference, metabolite damage and repair has been obtained through experiments and sagacity of researchers in analyzing these metabolic oddities. However, it is quite clear that if we could anticipate these complex problems early in the design of bioproduction pathway, or if we are able to predict new, more functional metabolic pathways by evolving promiscuous enzyme to be more efficient and specific to their novel substrate, we would make a great advance in systems and synthetic engineering by providing better performants and cost-effective microbial factories. This goal will require (i) collect a large set of data using bio foundry platforms able to screen a large collection of enzymes assayed against various substrates (ii) combine these experimental data and computational methods that relies on machine learning tools aiming at (i) predict catalytic promiscuity and substrate ambiguity and more importantly identify what structural properties of an enzyme account for its promiscuous activity (ii) underscore any potential beneficial of underground reactions and introduce them in genome-scale metabolic models and (iii) identify all metabolite damage and their repair mechanisms for optimal pathway efficiency in the microbial factory. All these questions provide great source of inspiration and opportunities for experimentalists and computer scientists alike in the near future.

## Author contributions

JMF drafted the manuscript, TM and CA managed the figures and all authors improved the final version.

## Funding

The work in JMF'lab in relation to this paper has been supported by French Research National Agency grant ANR-CE43-0008-01 and by grant EU-ERA CoBioTech n°ANR-21-COBOI-0003-01.

## Declaration of competing interest

The author declare that he has no competing interests.
